# Immunotherapy resistance in triple-negative breast cancer: Molecular mechanisms, tumor microenvironment, and therapeutic implications

**DOI:** 10.3389/fonc.2025.1630464

**Published:** 2025-08-27

**Authors:** Zijian Zhou, Qin Zhou

**Affiliations:** ^1^ School of Medicine, Jiangsu University, Zhenjiang, China; ^2^ Department of Breast Surgery, Affiliated Kunshan Hospital of Jiangsu University, Suzhou, China

**Keywords:** triple-negative breast cancer, drug resistance mechanism, genetic mutations, signaling pathways, personalized therapy, review literature

## Abstract

Triple-negative breast cancer (TNBC) is a unique subtype of breast cancer characterized by high invasiveness, high metastasis rates, and poor prognosis, making it an important focus within global malignancies. Due to the absence of estrogen receptor, progesterone receptor, and HER2 expression, TNBC presents significant challenges in treatment. Metastatic progression markedly increases treatment complexity, drastically reducing patient survival rates. The metastatic and drug resistance processes of TNBC involve complex, multi-step biological mechanisms regulated through various molecular mechanisms and signaling pathways within and outside tumor cells. In recent years, immunotherapy has brought new hope for TNBC. Compared to other breast cancer subtypes, TNBC demonstrates higher immunogenicity, often accumulating a higher mutational burden that generates more neoantigens, thus typically resulting in a tumor microenvironment (TME) enriched with tumor-infiltrating lymphocytes (TILs). Additionally, PD-L1 expression is significantly higher in TNBC compared to other subtypes, closely correlating with TIL abundance. These characteristics position TNBC as a strong candidate for immune checkpoint inhibitor (ICI) therapy. Clinical trials have demonstrated promising efficacy of ICIs in TNBC, overturning previous beliefs that breast cancer is generally insensitive to immunotherapy. This review summarizes recent advances regarding resistance types, molecular mechanisms, associated genes and pathways, the role of the tumor microenvironment, and clinical strategies related to immunotherapy resistance in the neoadjuvant setting of TNBC, aiming to provide insights and guidance for future research exploration and clinical practice.

## 1 Primary resistance and acquired resistance

Primary (Intrinsic) Resistance refers to the inherent insensitivity of tumor cells to therapeutic drugs at the initial stages of treatment, which occurs due to rapid adaptive capabilities of cancer cells or mutations in therapeutic targets. Such resistance typically arises from pre-existing genetic mutations or pathway abnormalities in cancer cells, making them inherently non-responsive to therapies. Approximately half of TNBC patients have substantial residual tumors (RCB II/III) after neoadjuvant chemotherapy (1), indicating poor initial treatment response. These patients exhibit a high risk of recurrence, reflecting the presence of primary resistance.

In contrast, acquired resistance develops when tumors gradually lose sensitivity to treatments that initially showed effectiveness. This form of resistance manifests during or after the treatment process. In TNBC, although about 32.6% of patients achieve a pathological complete response (pCR) following neoadjuvant chemotherapy (2), many experience rapid recurrence or metastasis soon after an initial positive response, indicating acquired resistance. Acquired resistance typically results from selective elimination of sensitive tumor cells by treatment, leading to the survival and proliferation of resistant cell clones. Mechanisms underlying acquired resistance include the emergence of new genetic mutations and feedback activation of signaling pathways.

Recent evidence indicates that approximately one-third of TNBC patients receiving combined neoadjuvant immunochemotherapy exhibit no pathological response, defined as primary resistance. Among patients who achieve initial remission, immunotherapy significantly prolongs event-free survival but does not entirely eliminate subsequent relapse risk, highlighting the issue of acquired resistance. Accurate identification of resistance phenotypes is critical for guiding therapeutic strategies: patients with primary resistance might benefit from prompt regimen adjustments or combinatorial therapeutic approaches, whereas individuals with acquired resistance require intensified adjuvant interventions to eradicate residual disease. Despite distinct underlying mechanisms, both forms of resistance profoundly limit therapeutic efficacy and necessitate deeper mechanistic exploration for precision-targeted interventions.

## 2 Molecular mechanisms of drug resistance

### 2.1 Increased drug efflux

ATP-binding cassette (ABC) transporters constitute a family of membrane transport proteins with significant biological functions and clinical implications. By harnessing energy from ATP hydrolysis, ABC transporters mediate the transmembrane transport of various substances, influencing numerous physiological and pathological processes. Drug-resistant tumor cells frequently exhibit overexpression of ABC transporters, which pump chemotherapeutic agents out of cells, thereby reducing intracellular drug concentrations. Members such as ABCB1 (P-glycoprotein or P-gp), ABCC1, and ABCG2 are directly implicated in multidrug resistance. In TNBC, ABCC1 and ABCG2 expression levels are notably higher compared to other breast cancer subtypes. For instance, P-gp is significantly elevated in paclitaxel-resistant breast cancer cell lines, and inhibiting its expression can partially restore drug sensitivity (3). Chemotherapy remains a cornerstone of neoadjuvant immunotherapeutic strategies for TNBC; consequently, overexpression of ABC transporters can significantly diminish chemotherapeutic efficacy by reducing tumor cell death and subsequent antigen release, thereby impairing downstream immune activation. To counteract this mechanism, researchers have explored pharmacological inhibition of ABC transporters utilizing non-steroidal anti-inflammatory drugs (NSAIDs), specific tyrosine kinase inhibitors, or downregulation through microRNA-mediated strategies, aiming to reverse multidrug resistance phenotypes (4). These adjunctive approaches may ultimately enhance responsiveness to combined chemo-immunotherapy regimens, representing a promising direction for future therapeutic refinement in TNBC.

### 2.2 Enhanced DNA damage repair

Abnormally activated DNA repair pathways are also key contributors to the development of drug resistance in TNBC. Many chemotherapeutic agents, such as platinum compounds and anthracyclines, exert cytotoxic effects by inducing DNA damage. However, TNBC cells with enhanced DNA repair capacity can efficiently repair chemotherapy-induced DNA lesions, thereby evading apoptosis and immune surveillance and ultimately acquiring resistance (5).Among the various repair mechanisms, the homologous recombination (HR) repair pathway plays a critical role, as it accurately repairs DNA double-strand breaks. Hyperactivation of HR can diminish the therapeutic efficacy of agents such as platinum-based drugs and poly(ADP-ribose) polymerase (PARP) inhibitors (6). Notably, Afghahi et al. demonstrated that although BRCA1/2-deficient TNBC is initially sensitive to DNA-damaging therapies, certain tumors may acquire secondary mutations that restore BRCA function, leading to resistance against platinum agents and PARP inhibitors (7).Efficient HR-mediated repair enables tumor cells to quickly resolve lethal DNA damage caused by chemotherapy or radiation, thereby avoiding elimination by both therapeutic agents and immune responses. Approximately 10%–20% of TNBC cases harbor mutations in HR-related genes—most commonly BRCA1 and BRCA2—rendering tumors HR-deficient and thus more sensitive to platinum-based chemotherapy and PARP inhibition. HR-deficient tumors tend to accumulate a higher mutational burden and generate more neoantigens, enhancing their immunogenicity and immune recognition. In contrast, BRCA wild-type TNBC maintains intact HR repair capacity, allowing effective DNA damage repair. This not only confers resistance to chemotherapy but also to immune-mediated cytotoxicity. Moreover, proficient HR repair reduces neoantigen production, decreasing tumor immunogenicity and contributing to intrinsic resistance to ICIs (8).

These mechanistic insights explain why BRCA-mutant (HR-deficient) TNBCs typically exhibit higher levels of TILs and are more responsive to immunotherapy, whereas HR-proficient TNBCs are often refractory to such treatments. As a result, evaluating HR repair status—such as through homologous recombination deficiency (HRD) scoring—has emerged as a crucial biomarker for guiding personalized therapeutic strategies in TNBC. In HR-proficient tumors, combining PARP inhibitors or other HR-targeting agents may impair DNA repair efficiency, thereby sensitizing tumors to immunotherapy and enhancing clinical outcomes.

### 2.3 Enhanced anti-apoptotic mechanisms (Bcl-2 family)

Cancer cells often upregulate anti-apoptotic proteins to counteract drug-induced cell death. The Bcl-2 protein family (including Bcl-2, Bcl-xL, Mcl-1) plays critical roles in mitochondrial apoptosis regulation (7). Overexpression of these proteins inhibits apoptotic signaling, promoting chemotherapy resistance. In TNBC, abnormal expression of Bcl-2 family proteins reduces chemotherapy-induced apoptosis, enabling tumor cell survival. Knockdown of Bcl-2 can increase chemosensitivity, and studies demonstrate that combining the Bcl-2 inhibitor ABT-199 with doxorubicin synergistically kills TNBC cells. These findings underscore the importance of Bcl-2-mediated anti-apoptotic mechanisms in TNBC drug resistance, suggesting targeted inhibition as a potential therapeutic strategy (9).

### 2.4 Increased autophagy activity (mTOR pathway regulation)

Autophagy is a cellular survival mechanism that recycles damaged components under stress conditions. Moderate autophagy helps maintain cellular homeostasis, whereas excessive autophagy facilitates tumor cell survival under chemotherapy. Elevated autophagy levels are frequently observed in TNBC, closely linked to acquired resistance (10). For example, sustained exposure to paclitaxel significantly enhances autophagy in TNBC cells; inhibiting autophagy (e.g., using chloroquine, CQ) decreases the IC50 of paclitaxel, restoring drug sensitivity. Autophagy regulation is tightly connected to mTOR activity: decreased mTOR activity triggers autophagy. Many anti-cancer treatments, such as nutrient deprivation or mTOR inhibitors, induce autophagy, thus aiding cancer cells in escaping drug effects. Autophagy elevation allows TNBC cells to survive chemotherapy by recycling damaged organelles and providing energy, forming an integral part of the resistance mechanism. Current studies targeting key autophagy regulators (e.g., eEF2K) explore reversing TNBC resistance (11).

### 2.5 Metabolic reprogramming (enhanced glycolysis)

Tumor cells frequently employ metabolic reprogramming to adapt to hostile environments and therapeutic stress. In TNBC, metabolic reprogramming not only fulfills the energetic demands required for rapid proliferation but also fosters an immunosuppressive microenvironment. A hallmark of such adaptation is the pronounced enhancement of glycolysis (the Warburg effect), leading to significant lactate production and subsequent extracellular acidification. This acidic microenvironment markedly inhibits the activity of effector T cells and natural killer (NK) cells, while concurrently promoting immunosuppressive regulatory T cell function. Lactate itself acts as a signaling molecule, interacting with the lactate receptor GPR81 on dendritic cells (DCs), thus suppressing antigen presentation and reducing the secretion of pro-inflammatory cytokines such as IL-6 and IL-12, thereby impairing T cell activation (12). Additionally, elevated lactate dehydrogenase (LDH) activity in tumor cells consumes glucose within the tumor microenvironment, causing energy deprivation in CD4^+^ T cells and elevating the expression of checkpoint molecules such as PD-1, consequently impairing their functionality (13).

A clinical study analyzing responses to neoadjuvant chemotherapy (NAC) in TNBC demonstrated that glycolytic pathways were significantly upregulated in tumors unresponsive to NAC, whereas responsive tumors exhibited relatively lower glycolytic activity. *In vitro* experiments also confirmed that paclitaxel treatment induces a metabolic shift in TNBC cells, enhancing their reliance on glycolysis while reducing mitochondrial oxidative metabolism (14). Importantly, inhibition of glycolysis enhances chemotherapy efficacy: blocking key glycolytic enzymes (such as hexokinase 2 with 2-deoxyglucose) significantly increases TNBC cell sensitivity to chemotherapy. Metabolic reprogramming thus provides TNBC cells with enhanced resilience and survival advantages, with glycolytic activation playing a pivotal role in resistance formation. Metabolism-targeted therapies, including glycolysis inhibition or lactate neutralization, may reshape the tumor microenvironment, enhance tumor immunogenicity, and improve sensitivity to immune checkpoint inhibitors, offering novel therapeutic strategies for overcoming immunotherapy resistance in TNBC.

## 3 Key genetic mutations associated with drug resistance

### 3.1 Tumor suppressor gene TP53

TP53 is a crucial tumor suppressor gene, and its encoded protein, p53, plays a central role in regulating the cell cycle, DNA repair, apoptosis, and cellular metabolism. In TNBC, TP53 mutations are highly prevalent, with mutation rates ranging from 65% to 80%. Molecular studies have demonstrated that TP53 mutations result in the loss of p53 function, thereby impairing the DNA damage response and enabling tumor cells to evade chemotherapy-induced apoptosis. This defect allows TNBC cells to tolerate the genomic damage inflicted by conventional chemotherapeutic agents, ultimately enhancing their survival (15).

To date, no targeted therapies specifically addressing p53 mutations have been approved for the treatment of breast cancer. However, significant efforts in clinical research are focused on restoring wild-type p53 activity through the reactivation of mutant p53. Among the most promising agents is APR-246, which has shown encouraging results in clinical trials. When combined with chemotherapy, APR-246 induced complete remission in approximately 47% of TP53-mutant patients with myelodysplastic syndrome (MDS) and acute myeloid leukemia (AML), providing a compelling rationale for translational application in breast cancer (16).

Furthermore, recent clinical observations have suggested that breast cancer patients harboring TP53 mutations may derive greater survival benefits from oncolytic virotherapy combined with paclitaxel (17). These findings imply that TP53 mutation status could serve as a predictive biomarker for identifying patients who are more likely to respond to immunotherapeutic strategies, offering new perspectives for personalized treatment in breast cancer.

### 3.2 Oncogene PIK3CA

PIK3CA mutations occur at a high frequency in various cancers and serve as important drivers of tumorigenesis. Lee et al. compared the mutational profiles between TNBC and HR+/HER2- breast cancer and found that the mutation rate of PIK3CA in TNBC was 8.3%, significantly lower than in HR+/HER2- breast cancer (36.4%), indicating a differential distribution across subtypes (18). PIK3CA encodes the p110α subunit of phosphatidylinositol 3-kinase (PI3K), a key molecule in the PI3K/Akt/mTOR signaling pathway, which regulates various cellular processes such as proliferation, growth, survival, and metabolism. Mutations in PIK3CA lead to aberrant activation of this pathway, promoting tumor cell proliferation and survival. Research by Huayu Hu et al. has shown that PIK3CA mutations confer chemotherapy resistance in TNBC by activating the PI3K/AKT/mTOR pathway and inhibiting apoptosis. PIK3CA-mutated TNBC cells exhibit reduced sensitivity to chemotherapy, along with enhanced proliferation and migration. In mouse models, tumors carrying the mutation grow faster and respond poorly to drugs (19). Domestic studies comparing the efficacy of α-subunit-targeted drugs, such as Inavolisib and Alpelisib, are currently in phase III clinical trials. These studies aim to assess the differences in efficacy when these drugs are combined with letrozole to treat HR+/HER2-/PIK3CA-mutated breast cancer patients who have developed resistance to CDK4/6 inhibitors and endocrine therapy. The results of these trials will provide high-level evidence for clinical treatment choices. The exploration of PI3K inhibitors combined with chemotherapy or immunotherapy may offer new pathways for overcoming chemotherapy resistance in PIK3CA-mutated TNBC.

### 3.3 Tumor suppressor genes BRCA1/2

BRCA1 and BRCA2 are essential tumor suppressor genes that play critical roles in the homologous recombination repair (HRR) pathway, responsible for the accurate repair of DNA double-strand breaks. Mutations in these genes compromise DNA repair capacity, thereby increasing the sensitivity of tumor cells to DNA-damaging agents. In TNBC, approximately 10% to 20% of patients carry BRCA1/2 mutations, which makes them particularly responsive to platinum-based chemotherapy and PARP inhibitors (20).

However, during treatment, some TNBC tumors may acquire secondary mutations that restore BRCA1/2 function, leading to resistance to platinum agents or PARP inhibitors. For instance, BRCA1 reversion mutations have been observed in TNBC patients following neoadjuvant platinum chemotherapy, which are associated with poor therapeutic outcomes and recurrence. Moreover, BRCA wild-type TNBC may compensate for homologous recombination deficiency by upregulating alternative DNA repair pathways, such as alternative lengthening of telomeres (ALT) or non-homologous end joining (NHEJ), thereby evading the effects of PARP inhibition (7).

Currently, PARP inhibitors remain the only gene-specific therapeutic option recommended by international guidelines for patients with BRCA1/2-mutated TNBC. Recently, Alradwan et al. developed an integrin-targeted nanoparticle system, iRGD-DOX-oHA-PLN, capable of co-delivering doxorubicin (DOX) and oligomeric hyaluronic acid (oHA). This platform synergistically inhibits key DNA repair factors, including RAD51 and PARP1, while markedly suppressing PD-L1 expression. In preclinical TNBC models, irrespective of BRCA mutation status, the iRGD-DOX-oHA-PLN formulation demonstrated superior antitumor efficacy and metastatic suppression compared to olaparib alone, underscoring its potential as a neoadjuvant immunotherapeutic strategy in BRCA-deficient tumors (21).

Further investigation into resistance mechanisms and the development of optimized combination strategies—such as PARP inhibitors with immune checkpoint inhibitors or ATR inhibitors—remains a vital direction for future TNBC research.

### 3.4 Androgen receptor

The androgen receptor (AR), a member of the nuclear steroid hormone receptor family, plays a crucial role as a transcription factor in signaling pathways and the regulation of gene expression. In TNBC cells, activation of the AR signaling pathway induces a phenotype similar to that of hormone receptor-positive breast cancers, with androgen-driven cell proliferation. Clinical studies have observed that AR-positive TNBC patients exhibit lower pathological complete response rates to standard chemotherapy and have relatively poor prognoses (22). This suggests that the AR signaling pathway may play a role in the poor chemotherapy response of TNBC, serving as a potential mechanism for suboptimal treatment outcomes. However, the aberrant activation of AR signaling also opens new avenues for treatment. Anti-androgen therapy has shown promise in clinical trials for AR-positive TNBC. Preclinical studies have demonstrated that inhibition of the AR can upregulate the expression of MHC class II molecules and tumor-associated antigens, thereby enhancing immune recognition of tumor cells and potentiating antitumor immune responses. In small-scale clinical trials, AR antagonists such as bicalutamide and enzalutamide have demonstrated some efficacy in treating AR-positive advanced TNBC patients (23).

### 3.5 Oncogene MYC

In TNBC, the proto-oncogene MYC is frequently amplified or aberrantly overexpressed. As a master transcription factor, c-MYC orchestrates key regulatory programs governing cell proliferation and metabolic adaptation. Extensive studies have established a strong correlation between c-MYC overexpression and the oncogenic progression, metastatic potential, and therapeutic resistance of TNBC, often portending a poor clinical prognosis. Elevated MYC levels not only induce the expression of pro-survival genes but also drive tumor cells toward a stem-like phenotype, thereby expanding the population of cancer stem cells (CSCs) and reprogramming glucose metabolism in favor of glycolysis (20). This metabolic shift enhances chemoresistance and diminishes treatment efficacy. Furthermore, MYC cooperates with anti-apoptotic proteins such as MCL-1 to amplify resistance to a broad spectrum of therapeutic agents (24).

Targeting MYC has thus emerged as a compelling strategy for overcoming drug resistance in TNBC. Recent mechanistic insights have revealed that breast cancer stem cells (BCSCs) can secrete macrophage migration inhibitory factor (MIF), which activates the WNT/β-catenin signaling axis, subsequently upregulating MYC and its downstream effector, the glycolytic enzyme ALDOC. This MIF–β-catenin–MYC–ALDOC cascade not only augments metabolic activity and lactate production but also fosters the establishment of an immunosuppressive microenvironment. Therapeutically, inhibition of MIF or downstream components of the MYC/ALDOC axis has been shown to attenuate glycolytic flux and, more importantly, to restore immune sensitivity. In preclinical models, blockade of this pathway synergistically enhances the efficacy of immune checkpoint blockade (e.g., anti-PD-1/PD-L1), as evidenced by increased infiltration of CD8^+^ cytotoxic T cells, and a concurrent reduction in Tregs and tumor-associated neutrophils (25).

MYC functions not only as a critical driver of resistance in TNBC but also as a molecular nexus linking oncogenic metabolism with immune evasion. Combinatorial approaches that co-target metabolic pathways and immune checkpoints represent a promising paradigm for the next generation of TNBC therapeutics (**Table 1**).

**Table 1:** Summary of major mechanisms of drug resistance in TNBC.

Mechanism	Key features	Representative molecules/pathways	Impact on therapy	Potential strategies
Increased Drug Efflux	Overexpression of ABC transporters	ABCB1, ABCC1, ABCG2	↓ intracellular drug concentration	ABC inhibitors, miRNA regulation
Enhanced DNA Repair	Efficient repair of chemo-induced DNA damage	BRCA1/2, RAD51, HR pathway	↓ sensitivity to platinum, PARPi	HRD-targeted agents, combination with ICIs
Anti-apoptotic Proteins	Upregulation of Bcl-2 family	Bcl-2, Mcl-1, Bcl-xL	Resistance to apoptosis	Bcl-2 inhibitors (e.g., ABT-199)
Autophagy Activation	Promotes survival under stress	eEF2K, mTOR pathway	↓ chemo efficacy	Autophagy inhibitors (e.g., CQ)
Metabolic Reprogramming	Enhanced glycolysis, lactate production	LDHA, MYC, GPR81	Immunosuppression, ↓ ICI response	Glycolysis inhibitors (e.g., 2-DG)
Key Genetic Alterations	TP53, PIK3CA, BRCA1/2, AR, MYC	See above	Altered therapy response	Targeted therapies, pathway inhibitors

## 4 The role of important signaling pathways in drug resistance

### 4.1 PI3K/AKT/mTOR pathway

The PI3K/AKT/mTOR signaling pathway is frequently and aberrantly activated in TNBC, serving as a central oncogenic driver of tumor progression and therapeutic resistance (26). This constitutive activation is commonly associated with gain-of-function mutations in PIK3CA. Persistent PI3K/AKT activation remodels the TIME into an immunosuppressive niche through multiple mechanisms. It upregulates VEGF, leading to abnormal angiogenesis and physical barriers that hinder T cell infiltration. Concurrently, AKT signaling induces chemokines such as CCL2, promoting the recruitment of immunosuppressive cells like tumor-associated macrophages (TAMs) and myeloid-derived suppressor cells (MDSCs) (27). Additionally, hyperactivated AKT impairs autophagy and effector T cell metabolism, weakening cytotoxic function and fostering T cell exhaustion (28). These alterations collectively dampen immune surveillance and contribute to ICI resistance, establishing PI3K/AKT hyperactivity as a key mechanism of immune evasion. Preclinical studies support the therapeutic potential of targeting this pathway. Inhibition of PI3K or AKT enhances T cell memory differentiation, persistence, and antitumor activity. In ICI-refractory TNBC models, dual blockade of the pathway increases CD8^+^ T cell infiltration and restores cytotoxicity, improving treatment outcomes (28). Moreover, PIK3CA-mutant TNBC cells exhibit sustained AKT/mTOR activation and upregulation of anti-apoptotic proteins such as Bcl-2 and XIAP, contributing to chemoresistance (19).

The PI3K/AKT/mTOR axis not only drives oncogenesis and drug resistance but also orchestrates immune evasion in TNBC. Targeting this pathway may enhance sensitivity to chemotherapy and immunotherapy, offering a compelling strategy for combination treatment in precision oncology.

### 4.2 JAK/STAT pathway (especially STAT3)

In TNBC, persistent activation of the JAK/STAT signaling pathway—particularly the STAT3 transcription factor—plays a central role in maintaining an immunosuppressive tumor microenvironment and driving resistance to therapy (29). Aberrant hyperactivation of the JAK2/STAT3 axis is frequently observed in TNBC and has been shown to transcriptionally upregulate anti-apoptotic genes such as Bcl-2 and Survivin, facilitate epithelial–mesenchymal transition (EMT), and promote tumor cell migration, invasion, and immune evasion. Moreover, STAT3 activation induces the expression of immunosuppressive cytokines like interleukin-10 (IL-10) and immune checkpoint molecules such as programmed death-ligand 1 (PD-L1), collectively suppressing effector T cell activity and promoting the expansion of regulatory T cells (Tregs). These changes contribute to a tumor-permissive immune microenvironment and diminished antitumor immune responses (30).To therapeutically target this pathway, Peng et al. (2024) developed a tumor-targeted delivery platform utilizing tumor-derived extracellular vesicles (TEVs) to co-deliver small interfering RNA against STAT3 (siSTAT3) and the chemotherapeutic agent doxorubicin (DOX), termed siSTAT3-DOX@TEV. This approach capitalizes on the inherent tumor-tropism of TEVs to achieve precise, dual-agent delivery. In both *in vitro* and *in vivo* models, the siSTAT3-DOX@TEV formulation effectively reduced intratumoral STAT3 expression and induced immunogenic cell death (ICD), thereby enhancing infiltration of CD4^+^ and CD8^+^ T cells and promoting polarization of macrophages toward the pro-inflammatory M1 phenotype. Concurrently, the proportion of immunosuppressive M2 macrophages was significantly reduced. Notably, expression of stromal markers such as CCL2 and α-SMA was also diminished, indicating successful remodeling of the tumor microenvironment and restoration of antitumor immunity (31).In addition to the STAT3-centric axis, the JAK1/2–STAT1 pathway is essential for mediating interferon-γ (IFN-γ) signaling, which is crucial for enhancing antigen presentation and initiating effective cytotoxic immune responses. However, genetic alterations that disrupt this signaling cascade—such as loss-of-function mutations in JAK2 or IRF1—can impair antigen presentation and contribute to resistance against immune checkpoint inhibitors (32). In TNBC specifically, non-functional JAK2 variants and deletions in B2M (β2-microglobulin) have been implicated as key mechanisms of acquired immune resistance (33).

The JAK/STAT signaling network exerts complex and multifaceted control over tumor immune evasion and therapeutic failure in TNBC. The TEV-mediated co-delivery strategy targeting STAT3 proposed by Peng et al. represents a promising and technically viable approach to mitigate STAT3-driven immunosuppression. These findings further highlight the translational potential of therapeutically modulating the JAK/STAT pathway as a central component of precision immunotherapy for TNBC.

### 4.3 Wnt/β-catenin pathway

The Wnt/β-catenin signaling pathway is frequently hyperactivated in TNBC, particularly within cancer stem cell–enriched subpopulations. This pathway exerts profound immunosuppressive effects through multiple, mechanistically distinct routes. Nuclear β-catenin promotes the transcription of MYC while concurrently suppressing the expression of major histocompatibility complex class I (MHC-I) molecules and the STING (stimulator of interferon genes) pathway, thereby attenuating antigen presentation and the secretion of chemoattractant cytokines (34). Additionally, β-catenin induces the transcription factor ATF3, which in turn downregulates CCL4 production, ultimately impeding the recruitment of CD103^+^ dendritic cells (DCs) and the subsequent activation and infiltration of CD8^+^ cytotoxic T cells (35). This spatial exclusion of T cells results in a non-inflamed tumor immune phenotype and is strongly associated with reduced responsiveness to ICIs.

Abreu et al. demonstrated, using both syngeneic TNBC models and patient-derived xenografts, that acquired resistance to platinum-based chemotherapy is accompanied by marked upregulation of Wnt/β-catenin signaling and concurrent enrichment of stemness-associated transcriptional programs. Importantly, pharmacological inhibition of Wnt signaling has been shown to restore sensitivity to carboplatin in resistant TNBC models, underscoring the therapeutic potential of targeting this pathway in overcoming drug resistance (36).

Further extending this mechanistic network, Taifour et al. (2023) identified a crosstalk between STAT3 and Wnt/β-catenin signaling. Their study revealed that STAT3 drives expression of its downstream effector Chi3l1, which facilitates neutrophil recruitment and formation of neutrophil extracellular traps (NETs), creating a dense extracellular matrix that acts as a physical barrier to T cell infiltration (37). This mechanism amplifies the immune-exclusion phenotype within the tumor, reinforcing resistance to ICIs.

Taken together, these findings position the Wnt/β-catenin and STAT3/Chi3l1 axes as cooperative regulators of immune evasion in TNBC. Their coordinated activity fosters a profoundly immunosuppressive microenvironment, and their dual inhibition may offer a rational combinatorial strategy to sensitize TNBC to immunotherapy.

### 4.4 RAS/MAPK/ERK pathway

The RAS–RAF–MEK–ERK pathway regulates cell proliferation and survival and is frequently hyperactivated in TNBC, particularly the basal-like subtype, through copy number gains in KRAS, BRAF, and RAF1, and NF1 loss, despite the rarity of RAS mutations (38).Elevated MAPK activity is inversely correlated with tumor-infiltrating lymphocyte (TIL) density and suppresses interferon-γ (IFN-γ) signaling. Constitutive RAS/MAPK activation reduces MHC class I/II and PD-L1 expression, impairing antigen presentation and CD8^+^ T cell function. These effects contribute to immune evasion and poor responses to ICIs in TNBC (39).Huang et al. demonstrated that epirubicin treatment induces transcriptional upregulation of MAPK/ERK pathway components and enhances ERK phosphorylation, suggesting a stress-induced survival mechanism. MEK/ERK inhibition may therefore sensitize TNBC cells to chemotherapy (40).Building on this, Ling Huang and colleagues identified BCAT1 as a covalent target of the natural compound Eupalinolide B (EB). EB binds to BCAT1’s catalytic Cys335, reduces intracellular leucine, and disrupts SHOC2 expression and its interaction with MRAS—thus suppressing RAS–ERK signaling (41).Pharmacologic BCAT1 inhibition reduced ERK phosphorylation, downregulated CCND1, CDK2, and PCNA, and upregulated Caspase-3 and TNFSF10. Leucine supplementation restored SHOC2 and ERK activation, highlighting a metabolic vulnerability in MAPK-driven TNBC.

### 4.5 NF-κB pathway

NF-κB is a key pro-survival and pro-inflammatory signaling axis that exhibits constitutive activation in TNBC, with expression markedly elevated relative to normal mammary tissue (42). Its activation promotes transcription of anti-apoptotic and oncogenic effectors such as BCL-xL, XIAP, Survivin, and IL-6, thereby enhancing tumor cell survival, proliferation, and chemoresistance. The hypoxic tumor microenvironment further amplifies NF-κB signaling, contributing to therapy resistance and facilitating pro-tumorigenic inflammation and angiogenesis. Persistent NF-κB activity has been consistently linked to poor prognosis in TNBC (43).Given its central role in resistance, NF-κB represents a promising therapeutic target. However, systemic inhibition poses risks of toxicity and immune dysfunction due to its physiological roles (44). To address this, recent strategies have utilized nanomedicine for selective pathway modulation. One study developed a multifunctional self-assembling nanoparticle (VHEB) incorporating a chloroquine derivative and bortezomib (BTZ), an NF-κB antagonist. This formulation enabled tumor-specific delivery, inhibited NF-κB nuclear translocation, reduced IL-6 secretion, and mitigated immunosuppression. VHEB also induced immunogenic cell death (ICD), promoting antigen-presenting cell activation and CD8^+^ T cell infiltration. In preclinical TNBC models, this approach enhanced the efficacy of anti–PD-L1 therapy, improving tumor control and survival (45). These findings highlight the potential of nanoparticle-mediated NF-κB modulation to sensitize TNBC to immunotherapy and support its integration into targeted immuno-metabolic treatment strategies.

### 4.6 Multi-pathway interactions in drug resistance

**Figure 1:**
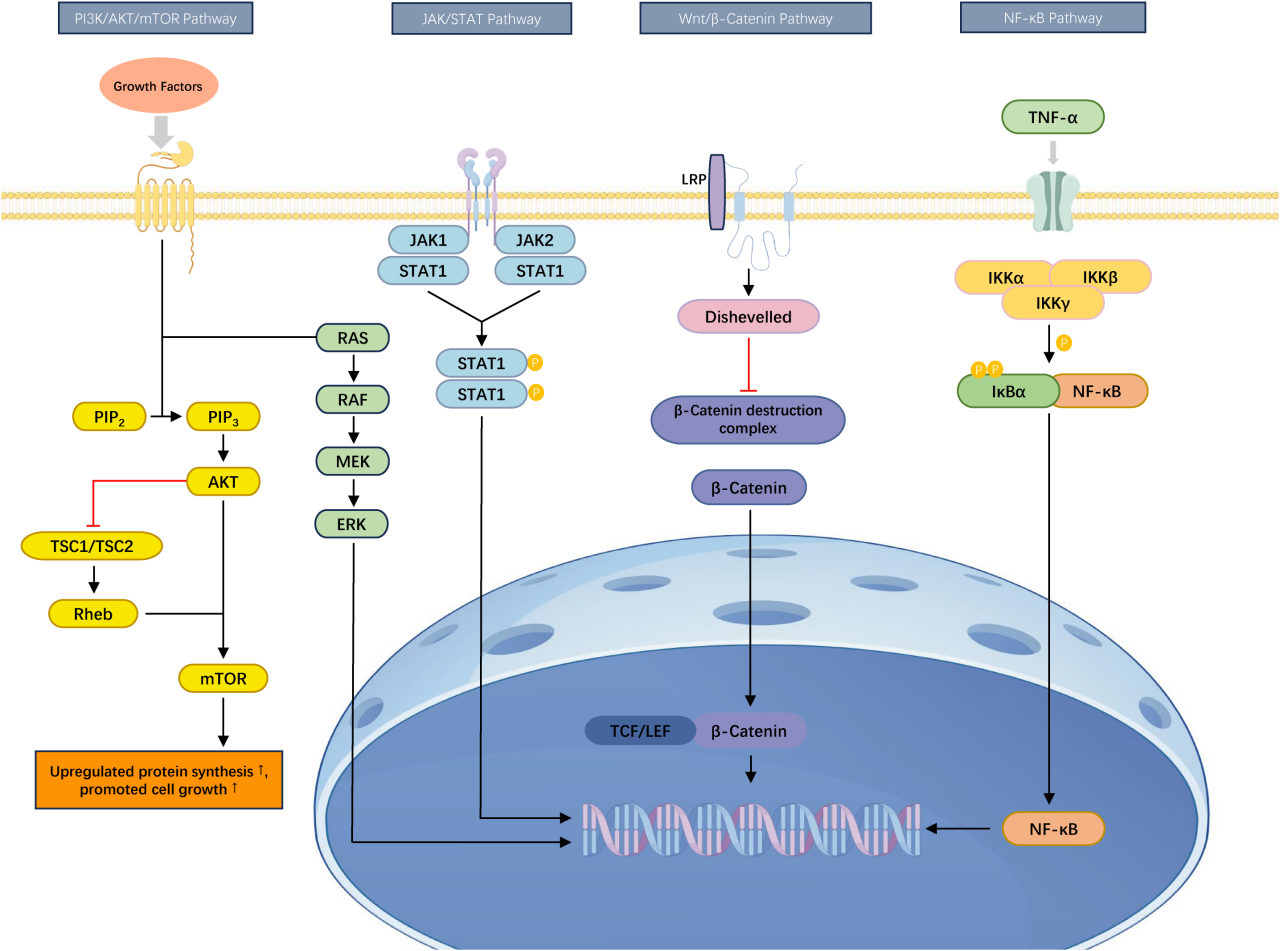
Key signaling pathways associated with drug resistance in TNBC. The figure illustrates the PI3K/AKT/mTOR, JAK/STAT, Wnt/β-catenin, and NF-κB signaling pathways, which are closely linked to drug resistance and immune evasion in TNBC. These pathways regulate critical cellular processes including proliferation, apoptosis inhibition, immune suppression, and metabolic reprogramming, thereby contributing to both intrinsic and acquired therapeutic resistance.

TNBC drug resistance is not solely the result of isolated pathways but involves complex interactions among multiple signaling pathways. For instance, in PTEN-deficient TNBC subtypes, the activation of the PI3K/AKT/mTOR pathway synergistically amplifies signals from Wnt/β-catenin and MYC, forming a feedback loop that enhances resistance. AKT phosphorylates and inhibits GSK3β, stabilizing β-catenin and promoting the expression of Wnt target genes, while MYC, as a common downstream target of both PI3K and Wnt signaling, drives cell cycle progression and metabolic reprogramming. These interactions generate multiple layers of resistance barriers. Additionally, the JAK/STAT and NF-κB pathways exist in dynamic balance. JAK inhibitors may block STAT3 signaling, but they can inadvertently activate NF-κB in macrophages, leading to the secretion of pro-survival factors such as IL-6 and CSF2, which activate tumor cell STAT3 and PI3K signaling through paracrine mechanisms, forming an escape loop from treatment. These inter-pathway interactions suggest that targeting a single pathway may lead to compensatory resistance, and future strategies should focus on developing combination therapies that target multiple pathway nodes for more effective overcoming of TNBC resistance (**Figure 1**).

## 5 The role of tumor microenvironment in drug resistance

### 5.1 PD-L1-mediated immune evasion

TNBC often exhibits significant immune cell infiltration, yet tumor cells evade immune-mediated cytotoxicity through upregulation of immune checkpoint molecules like PD-L1. Expressed on both tumor and tumor-associated immune cells, PD-L1 binds PD-1 on T cells to suppress their activation and effector function, reducing antitumor immunity. High PD-L1 expression is associated with reduced response to immune checkpoint blockade and reflects intrinsic immunotherapy resistance. Clinical trials have shown that combining the anti–PD-L1 antibody atezolizumab with chemotherapy improves progression-free and overall survival in patients with PD-L1–positive metastatic TNBC, leading to its approval for this subset (46). However, PD-L1–negative patients show limited benefit. Beyond its immune checkpoint function, PD-L1 contributes to resistance via intracellular signaling. Wofford et al. found that CerS4 downregulation promotes PD-L1 internalization without altering total expression. Internalized PD-L1 forms a complex with caprin-1, stabilizing metastasis-associated mRNAs (e.g., Shh, TGFBR1, Wnt components) and activating β-catenin signaling, thereby enhancing migration and resistance. CerS4 loss also correlates with increased FoxP3^+^ regulatory T cells and T cell exhaustion. Co-treatment with the Shh inhibitor sonidegib and anti–PD-L1 antibody blocks PD-L1 internalization and downstream signaling, reducing tumor progression and improving CD8^+^ T cell/Treg balance in preclinical models (47). Complementing these findings, Cao et al. identified CREB3L2—a noncanonical UPR factor—as a paracrine regulator of immune evasion in TNBC. Its cleaved C-terminal fragment activates Hedgehog signaling in CD8^+^ T cells, suppressing their cytotoxicity and promoting resistance to PD-1 blockade. Inhibiting the Hedgehog pathway reactivates T cell function and restores immunotherapy sensitivity in CREB3L2-high tumors (48). This highlights an additional immune escape mechanism independent of PD-L1 expression, driven by tumor stress signaling.

These findings reveal that immune escape in TNBC involves both canonical PD-L1–mediated inhibition and noncanonical, secreted factor–driven suppression. Targeting PD-L1 trafficking and UPR-linked signals like CREB3L2 may enhance immunotherapy efficacy.

### 5.2 Cancer-associated fibroblasts

Cancer-associated fibroblasts (CAFs), as essential components of the tumor microenvironment (TME), significantly contribute to therapeutic resistance in TNBC. In addition to promoting chemoresistance, CAFs facilitate immune evasion and impair responses to immune checkpoint inhibitors (ICIs). By secreting factors such as TGF-β and HGF, CAFs activate survival pathways, drive epithelial–mesenchymal transition (EMT), and enhance tumor progression. Recent findings by Wall et al. revealed that CAF-derived signals promote TNBC cell proliferation during chemotherapy intervals, accelerating tumor regrowth. This paradoxical effect, wherein CAFs support proliferation even under chemotherapeutic pressure, may reduce the complete response (CR) rate by fostering residual tumor survival between treatment cycles (49).CAFs also remodel the extracellular matrix (ECM), increasing stromal density and rigidity, which limits T cell infiltration and impairs ICI efficacy, particularly PD-1/PD-L1 blockade. The immunosuppressive tumor immune microenvironment (TIME), dominated by the myofibroblastic CAF (myoCAF) subset, poses a major barrier to immunotherapy. MyoCAFs, marked by high α-SMA and FAP expression, reinforce fibrotic stroma and engage in inhibitory interactions such as the PDCD1–FAM3C axis, suppressing T cell activity (50). Wu et al. identified elevated FGFR signaling in immune-excluded TNBC, predominantly within CAFs. FGFR activation promotes CAF proliferation, migration, and VCAM-1 secretion, forming barriers that block T cell infiltration. Erdafitinib inhibited the MAPK/ERK pathway in CAFs, reduced VCAM-1, disrupted stromal architecture, and enhanced CD8^+^ T cell entry, thereby improving anti–PD-1 response (51). Huaier, a traditional Chinese medicine, was found to inhibit TGF-β/SMAD signaling, block myoCAF differentiation, remodel the TIME, enhance CD8^+^ T cell infiltration and cytotoxicity, and synergize with anti–PD-1 therapy in preclinical models (52).

Targeting specific CAF subpopulations, particularly myofibroblastic CAFs (myoCAFs), offers a promising approach to reprogram the immunosuppressive tumor microenvironment, limit tumor repopulation after chemotherapy, and enhance the response to immunotherapy in TNBC.

### 5.3 Angiogenesis (VEGF-mediated)

In the tumor microenvironment (TME), abnormal vasculature and the resulting hypoxic and acidic conditions are key contributors to immune tolerance. TNBC is typically characterized by active angiogenesis; however, the newly formed blood vessels are often structurally abnormal and poorly perfused, leading to persistent intratumoral hypoxia. Hypoxia induces transcription factors such as HIF-1α, which not only promote a more invasive tumor phenotype but also upregulate various immunosuppressive molecules, including PD-L1 and pro-angiogenic factors (53).

Furthermore, hypoxia drives tumor cells and tumor-associated macrophages to highly express ectoenzymes such as CD39 and CD73 on their surfaces. These enzymes catalyze the conversion of extracellular ATP into high levels of adenosine, which in turn acts on A2A receptors on immune cells to broadly suppress the activity of effector T cells and natural killer (NK) cells, while simultaneously promoting the development of regulatory T cells (Tregs) (12). This constitutes the immunosuppressive hypoxia–adenosine axis. In addition, as previously mentioned, hypoxia-induced accumulation of lactic acid further impairs immune responses.

Abnormal tumor vasculature also contributes to therapeutic resistance through two key mechanisms: first, the vascular barrier hinders immune cell infiltration from the bloodstream into tumor tissues; second, elevated interstitial pressure caused by aberrant vasculature restricts lymphocyte migration and retention within the tumor. Given the central role of tumor vasculature and hypoxia in immune evasion, anti-angiogenic therapies have been proposed as a means to synergize with immunotherapy. Anti-VEGF antibodies (e.g., bevacizumab) can transiently “normalize” tumor vasculature, reducing permeability and improving perfusion, thereby enhancing T cell infiltration and function (27).

**Figure 2:**
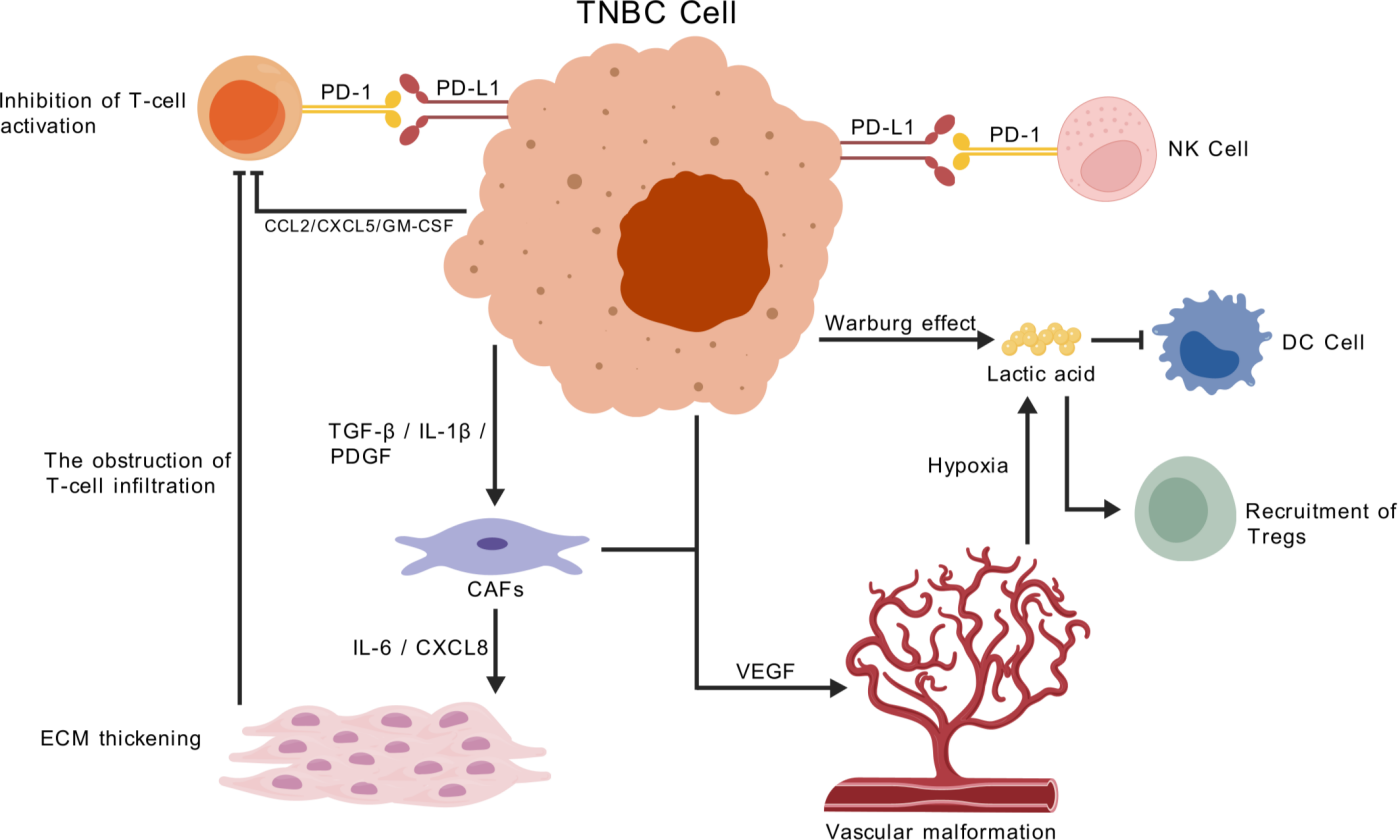
Immunosuppressive tumor immune microenvironment (TIME) in TNBC. The schematic illustrates major immunosuppressive mechanisms in TNBC. Tumor cells secrete PD-L1, lactate, adenosine (via CD73), VEGF, and TGF-β, largely driven by hypoxia-induced HIF-1α. These signals suppress CD8^+^ T cells, NK cells, and dendritic cells. Regulatory T cells (FoxP3^+^), MDSCs, and TAMs further inhibit immunity via cytokines and metabolic pathways. CAFs release VEGF and CXCL12, forming a fibrotic barrier that blocks immune infiltration. Together, these interactions create a “cold” TIME and contribute to immune checkpoint therapy resistance.

VEGF-mediated angiogenesis in TNBC fosters an immunosuppressive microenvironment by limiting immune cell infiltration and promoting regulatory immune cells (54). Targeting the VEGF pathway may normalize tumor vasculature and improve immune accessibility, thereby enhancing the efficacy of immune checkpoint inhibitors. A phase II trial combining tislelizumab, bevacizumab, and nab-paclitaxel demonstrated a 73.3% objective response rate in metastatic TNBC, including PD-L1–negative patients, highlighting the synergistic potential of anti-angiogenic and immunotherapy strategies (**Figure 2**) (55).

## 6 Clinical strategies for overcoming TNBC drug resistance

### 6.1 Combination therapy strategies

Combination therapies that target multiple aspects of drug resistance have shown promise in improving treatment outcomes for TNBC. By combining therapies with different mechanisms of action, these strategies can simultaneously target various resistance mechanisms, reducing the chances of surviving resistant clones. One promising approach is combining chemotherapy with immunotherapy. For instance, a phase III trial of Atezolizumab (anti-PD-L1 antibody) combined with nab-paclitaxel in metastatic TNBC demonstrated significant improvements in progression-free survival and overall survival, particularly in PD-L1-positive patients. Based on these findings, chemotherapy combined with anti-PD-1/PD-L1 therapy has been incorporated into clinical guidelines for advanced TNBC. Another approach is combining chemotherapy with targeted therapy: in cases where specific pathways are aberrantly activated, the addition of targeted drugs can enhance the effect. For example, in basal-like 2 subtype TNBC, the EGFR pathway is often active. Research has shown that combining anti-EGFR monoclonal antibodies (e.g., cetuximab) with chemotherapy improves progression-free survival and overall survival in TNBC patients. Similarly, for patients with PI3K/AKT pathway abnormalities, combining chemotherapy with AKT inhibitors, or using MEK inhibitors in cases with RAS/MAPK pathway mutations, is being tested in clinical trials (56). By targeting multiple pathways, these combination strategies aim to overcome the limitations of single-agent treatments and improve overall therapeutic efficacy.

### 6.2 New drug applications

Several new drugs have been introduced to treat TNBC, with PARP inhibitors standing out as a notable class. These inhibitors have been particularly effective in patients with BRCA mutations or homologous recombination deficiencies. Clinical trials have shown that single-agent PARP inhibitors, such as Olaparib and Talazoparib, significantly extend progression-free survival in these patients and have been approved by the FDA for the treatment of advanced breast cancer with germline BRCA mutations (57). Immune checkpoint inhibitors, such as Pembrolizumab (anti-PD-1), are also being used in early-stage high-risk TNBC in neoadjuvant and adjuvant settings, significantly increasing the pathological complete response (pCR) rate and invasive disease-free survival (20). In the KEYNOTE-522 study, the addition of Pembrolizumab to neoadjuvant chemotherapy increased the pCR rate by nearly 15% in TNBC. These new drugs provide novel options for treating refractory TNBC. Additionally, antibody-drug conjugates (ADCs) like Sacituzumab Govitecan (an anti-Trop-2 monoclonal antibody conjugated to a topoisomerase inhibitor) have been approved for second-line treatment of metastatic TNBC, showing objective responses even in patients who have failed prior therapies. Androgen receptor antagonists are being explored for AR-positive TNBC, and PI3K/mTOR inhibitors are under investigation for TNBC with PIK3CA mutations or PTEN loss.

### 6.3 Selecting the appropriate timing for immunotherapy

In triple-negative breast cancer (TNBC), resistance to immunotherapy is not only associated with intrinsic tumor characteristics but is also significantly influenced by the timing of treatment. Clinical evidence suggests that the sequence of immunotherapy administration may impact treatment response. In the phase III CamRelief trial, Chen et al. employed camrelizumab in combination with chemotherapy as neoadjuvant treatment, followed by postoperative maintenance in some patients. The addition of immunotherapy significantly improved pathological complete response (pCR) rates, reinforcing the therapeutic value of incorporating immunotherapy in the preoperative phase (58). In contrast, the ALEXANDRA/IMpassion030 trial reported by Ignatiadis et al. investigated the use of atezolizumab in the adjuvant setting, following surgery and chemotherapy, but failed to demonstrate an improvement in invasive disease-free survival (iDFS). This suggests that initiating immunotherapy postoperatively may be insufficient to adequately stimulate antitumor immune responses, further highlighting the critical role of treatment timing (59).

As an emerging class of targeted agents, antibody-drug conjugates (ADCs) also demonstrate timing-dependent synergy with immunotherapy. In TNBC, early integration of ADCs with immunotherapy may be more effective in delaying the onset of resistance, whereas later-line administration may be limited by the development of tumor-acquired resistance (60). Strategically optimizing the timing of immunotherapy—particularly during the neoadjuvant phase—may be key to maximizing immune activation and overcoming resistance. Future studies should further explore biomarker-driven approaches to individualized treatment and systematically assess how different immunotherapy timing strategies affect long-term survival outcomes, in order to refine therapeutic decision-making for patients with TNBC.

### 6.4 The application of nanotechnology

In the treatment of triple-negative breast cancer (TNBC), nanotechnology has garnered increasing attention due to its unique advantages in drug delivery, targeted therapy, and modulation of the tumor microenvironment. Fan et al. designed a tri-component micro-nano composite system, CS-6@CPB-S.lux, which integrates gamabufotalin (CS-6), known for its anti-PD-L1 activity, with photothermal carboxy-Prussian blue nanoparticles (CPB NPs) and a tumor-tropic attenuated Salmonella typhimurium strain (S.lux) into a single platform. This system induces immunogenic cell death (ICD) through combined photothermal and chemotherapeutic mechanisms, promotes dendritic cell maturation and effector T cell activation, and facilitates polarization of tumor-associated macrophages while modulating the immune microenvironment. These synergistic effects inhibit both primary and metastatic TNBC lesions and significantly prolong survival time (61). Gao et al. developed a nanoplatform, DD@FEL, co-loaded with doxorubicin and doxycycline, employing a combinatorial approach to exert both cytotoxic and antimicrobial effects. This system selectively eradicates intratumoral Staphylococcus and Lactobacillus species enriched in breast tumors, reduces bacteria-induced cell migration and lung metastasis potential, and thereby delays TNBC progression (62).Ma et al., in their investigation of targeted nanoprobe applications in TNBC, emphasized that active targeting strategies—such as surface modifications with antibodies, peptides, or saccharides—can recognize overexpressed receptors specific to TNBC cells, including CD44 and αvβ3 integrin, thereby enhancing nanoparticle accumulation in tumor tissues. Representative studies include polymeric fluorescent probes modified with RGD peptides, supramolecular drug conjugates functionalized with RGDR, and HA/CD44-based nanogels. These platforms demonstrate strong targeting capability, minimal side effects, and stable therapeutic performance in terms of controlled drug release, immune response activation, and anti-tumor efficacy (63).

### 6.5 Personalized precision therapy

Due to the high heterogeneity of TNBC, the mechanisms of drug resistance vary among patients, making personalized treatment strategies essential. Identifying the molecular subtype of TNBC and designing a personalized therapeutic approach is critical. Artificial intelligence-based diagnostic systems, which analyze structural features from pathology or imaging data, can rapidly and non-invasively classify molecular subtypes. The “one-stop” online decision support platform for breast cancer, developed by institutions such as Fudan University, has already achieved clinical translation, providing efficient and precise diagnostic and treatment services in regions like the Yangtze River Delta (64).

Genetic testing and molecular profiling help formulate targeted treatment plans. For example, patients with the Basal-like subtype, which is more sensitive to platinum-based chemotherapy and PARP inhibitors, may be prioritized for these drugs. For LAR subtype TNBC, which is driven by AR expression, anti-androgen therapy should be considered. The Mesenchymal subtype, rich in EMT and stem cell characteristics, may benefit from inhibitors targeting pathways such as TGF-β, Notch, or Wnt. Additionally, mutations like PIK3CA can be targeted with PI3K inhibitors, and BRCA mutations may benefit from PARP inhibitors. For tumors with high microsatellite instability or a high mutational load, immunotherapy could be an option. The trend is moving from “one-size-fits-all” approaches toward more tailored, individualized treatments. Although personalized therapy for TNBC faces challenges (e.g., lack of validated predictive biomarkers), integrating multi-omics data and real-time monitoring of resistant clones may allow for earlier adjustments to therapy and help overcome drug resistance.

The mechanisms underlying drug resistance in triple-negative breast cancer (TNBC) are multifaceted, involving intrinsic genetic alterations, dysregulation of signaling pathways, and the protective influence of the tumor microenvironment. This review synthesizes current knowledge of TNBC resistance, with a particular focus on tumor-intrinsic factors, immune evasion mechanisms, and therapeutic vulnerabilities. While the analysis provides a broad and timely overview, several limitations should be acknowledged. As a narrative review, the study does not employ a systematic methodology for literature selection or evidence appraisal, which may introduce selection bias and overlook emerging but underrepresented studies. Moreover, the absence of quantitative data—such as the prevalence of specific resistance mechanisms across molecular subtypes or treatment contexts—limits the translational applicability of the findings. The review also does not incorporate high-dimensional data sources, including single-cell transcriptomics, spatial profiling, and proteogenomics, which are increasingly essential for dissecting intratumoral heterogeneity and tumor–immune interactions. Although therapeutic strategies are discussed, the connection between mechanistic insights and clinical decision-making remains largely conceptual, lacking critical evaluation of biomarker-guided interventions and ongoing clinical trials. These limitations underscore opportunities for future refinement in both the scope and methodological rigor of resistance-focused research in TNBC.

Continued investigation into these resistance mechanisms is progressively informing the development of more effective therapeutic strategies, including rational combination regimens, next-generation targeted agents, and personalized treatment approaches tailored to the molecular and immunological landscape of individual tumors. As clinical evidence accumulates and novel therapies emerge, meaningful advances are expected in overcoming resistance and improving the outcomes of patients with this aggressive breast cancer subtype.
